# Color-Guided Depth Map Super-Resolution Using a Dual-Branch Multi-Scale Residual Network with Channel Interaction

**DOI:** 10.3390/s20061560

**Published:** 2020-03-11

**Authors:** Ruijin Chen, Wei Gao

**Affiliations:** 1 National Laboratory of Pattern Recognition (NLPR), Institute of Automation, Chinese Academy of Sciences, Beijing 100190, China; ruijin.chen@nlpr.ia.ac.cn; 2 School of Artificial Intelligence, University of Chinese Academy of Sciences, Beijing 100049, China

**Keywords:** depth map, super-resolution, guidance, residual network, channel interaction

## Abstract

We designed an end-to-end dual-branch residual network architecture that inputs a low-resolution (LR) depth map and a corresponding high-resolution (HR) color image separately into the two branches, and outputs an HR depth map through a multi-scale, channel-wise feature extraction, interaction, and upsampling. Each branch of this network contains several residual levels at different scales, and each level comprises multiple residual groups composed of several residual blocks. A short-skip connection in every residual block and a long-skip connection in each residual group or level allow for low-frequency information to be bypassed while the main network focuses on learning high-frequency information. High-frequency information learned by each residual block in the color image branch is input into the corresponding residual block in the depth map branch, and this kind of channel-wise feature supplement and fusion can not only help the depth map branch to alleviate blur in details like edges, but also introduce some depth artifacts to feature maps. To avoid the above introduced artifacts, the channel interaction fuses the feature maps using weights referring to the channel attention mechanism. The parallel multi-scale network architecture with channel interaction for feature guidance is the main contribution of our work and experiments show that our proposed method had a better performance in terms of accuracy compared with other methods.

## 1. Introduction

With the development of 3D technologies, such as 3D reconstruction, robot interaction, and virtual reality, the acquisition of precise depth information as the basis of 3D technology has become very important. At present, depth maps can be obtained conveniently using low-cost depth cameras. However, depth maps obtained under such hardware constraints are usually of low resolution. To use low-cost depth maps in 3D tasks, we need to perform super-resolution (SR) processing on low-resolution (LR) depth maps to obtain high-resolution (HR) depth maps.

The main difficulty of depth map SR tasks is that the spatial downsampling of HR images to LR images will result in the loss and distortion of details, and this phenomenon will become more serious as the downscaling factor increases. When we want to recover HR images from LR images using simple upsampling, an edge blur and other detail distortion problems will appear. To cope with these problems, methods of using HR intensity images to guide the upsampling process of LR images have been proposed. The realization of these methods is based on the corresponding association relationship between HR intensity images and LR depth maps in the same scene. If the resolution of intensity image and target HR depth map are the same, edges of the intensity image and the target HR depth map can be regarded as basically corresponding, and therefore discontinuities in the intensity image can help to locate discontinuities in the target HR depth map during upsampling on the LR depth map. Although the introduction of intensity image guidance during the upsampling process will alleviate the blur of details like edges, extra textures may be introduced into the generated HR depth map owing to the inconsistency of the structure between the depth map and the intensity image.

We proposed an end-to-end, multi-scale deep map SR network, which consists of two branches, namely the RGB image branch (Y-branch) and the depth map branch (D-branch). Each branch is mainly composed of residual levels at multiple scales, and each residual level has two functional structures of feature extraction and upsampling. Among them, feature extraction is achieved by connecting several residual groups, each of which contains several residual blocks. As the key to residual structure, the internal short-skip connections of residual blocks and the long-skip connections in residual groups and levels enable the main road of branch network to learn the high-frequency information of the RGB image or depth map at different scales. Feature extraction parts in every residual level correspond one-to-one, which means that channel-wise, high-frequency features learned by each residual block of the Y-branch can be input into the corresponding residual block of the D-branch. On this foundation, we utilized a channel attention mechanism to rescale the channel-wise feature maps and fuse these features from two branches to implement guidance from the RGB image to the depth map. Under this kind of guidance, features in the HR depth map are supplemented, meanwhile weights in the aforementioned channel-wise feature rescaling limits the addition of artifacts from the RGB image. Compared with many existing methods, we input the LR depth map and HR RGB image directly into the network instead of inputting a bicubic interpolation of the LR depth map. Experiments indicate that our proposed method achieved great performances when recovering an HR depth map from an LR depth map with different upscaling factors.

The main contributions of our work are:

1. We designed a multi-scale residual network with two branches to realize an end-to-end LR depth map super-resolution under the guidance from an HR color image.
2. We applied a channel attention mechanism [1] to learn the features of a depth map and RGB image and fuse them via weights; furthermore, we tried to avoid copying artifacts to the depth map while ensuring the guidance from RGB image worked.
3. We discuss the detailed steps toward realizing image-wise upsampling and end-to-end training of this dual-branch, multi-scale residual network.

## 2. Related Works

There have been many methods proposed to complete the task of depth map SR reconstruction. Based on whether the method uses the guidance of an intensity image, the methods for depth map super-resolution can be divided into two categories, namely methods only based on depth maps and methods based on depth maps and intensity images.

Regarding methods based on depth maps, some methods are based on filters. The filter-based methods calculate the depth value of a pixel using its local information. Narayanan et al. [2] proposed a modified adaptive Wiener filter and a spatially adaptive signal-to-noise ratio estimate for reconstructing HR JPEG2000-compressed images. Lu et al. [3] used image segmentation and proposed a smoothing method to reconstruct the depth structure of each segmentation. Some methods are based on a dictionary that employs the relationship between each patch pair of LR and HR depth maps through sparse coding. Kwon et al. [4] defined an upscaling problem and introduced a scale-dependent dictionary. Xie et al. [5] proposed a framework that reconstructs a depth map’s edge firstly and then reconstructs the HR depth map. These methods based on a dictionary usually require image block extraction and pre-processing operations that are difficult to implement for an end-to-end image super-resolution. In addition, it is hard to establish correct mapping between LR and HR image blocks in the dictionary. Some methods are based on a convolution neural network (CNN) and differ from dictionary-based methods by not explicitly learning a mapping dictionary. Dong et al. [6] proposed an SR reconstruction method called a super-resolution convolutional neural network (SRCNN) based on a CNN, which uses three convolution layers to non-linearly map a LR feature space to a HR feature space. This network has a relatively simple structure and small receptive fields such that it can only learn a few features. Kim et al. [7] proposed a VDSR (Very Deep Super Resolution) network that has 20 layers and learns more features. VDSR pre-processes the input depth map using bicubic interpolation that affects the network’s learning of the LR depth map’s original information and introduces artifacts to the reconstructed HR depth map. Lai et al. [8] proposed a Laplacian pyramid SR network called LapSRN that gradually reconstructs the sub-band residuals of HR images and uses transposition convolution to generate HR images. The input of LapSRN is an LR image without bicubic interpolation such that artifacts can be avoided. However, checkerboard artifacts [9] will occur if network parameters, such as the kernel size, are set improperly.

Regarding methods based on depth maps and intensity images, some methods are based on filters. He et al. [10] enhanced an LR depth map by assuming a linear relationship between the patches of the image for guidance and the output depth map. Barron and Poole [11] proposed a fast bilateral solver that can be used for enhancing the depth map under the guidance from a color image. Some methods are based on optimization. In these methods, depth upsampling is defined as an optimization problem in which if a pixel’s neighboring pixels have similar colors in the intensity image but different values in the depth map, then this pixel will be given a large loss value and the total loss of all pixels needs to be minimized. Diebel et al. [12] proposed a MRF (Markov Random Fields) formula containing a data term from an LR depth map and a smooth term from an HR intensity image. Park et al. [13] integrated edge, gradient, and segmentation from an HR color image to design the anisotropic affinities of the regularization terms. Ferstl et al. [14] used a secondary generalized variable guided by an anisotropic diffusion tensor extracted from an HR color image to limit a regularized HR depth map. Zuo et al. [15,16] measured the discontinuities of edges between a color image and a depth map in an MRF, and these discontinuities can be reflected in the edge weight of the minimum spanning tree. Yang et al. [17] proposed a novel depth map SR method guided by a color image by using an auto-regression model. All these optimization-based methods are based on the assumption that the edges of a color image and a depth map have consistency. However, textures in a color image may not have corresponding regions in a depth map, which will override the assumption of consistency and introduce artifacts to the reconstructed HR depth map. Some methods are based on a dictionary. Kiechle et al. [18] proposed a dual-mode co-sparse analysis model that reconstructs a depth map by capturing the interdependence between the intensity of a color image and the depth of a depth map. Some methods are based on a CNN. Riegler et al. [19] designed a kind of special end-to-end deep convolution neural network (DCNN) to learn data terms and regulation terms in an MRF that reconstructs an HR depth map. Zhou et al. [20] developed a new DCNN to jointly learn nonlinear projection equations when noise occurs. Yang et al. [21] learned joint features to obtain an HR depth map guided by the edge attention map extracted from an HR color image. Ye et al. [22] designed a kind of DCNN to learn the binary map of depth edge positions from an LR depth map under the guidance of a corresponding HR color image. These DCNNs introduce noise to the output HR depth map by inputting the interpolated LR depth map, which is ineffective for processing features in the high-frequency domain. Hui et al. [23] proposed a DCNN that accepts multi-scale guidance from an HR intensity image and mainly learns features in the high-frequency domain. Zuo et al. [24] proposed a data-driven approach based on a CNN with local residual learning introduced in each scale-dependent reconstruction sub-network and global residual learning is utilized to learn the difference between the upsampled depth map and the ground truth. Zuo et al. [25] proposed a DCNN to reconstruct the HR depth map guided by the intensity image, where dense connections and sub-networks recover the high-frequency details from coarse to fine. These DCNNs adopt a residual network or multi-scale upsampling mechanism like our proposed network but the ways in which the intensity image guides the process are different, which determines a difference in the severity of artifacts. Voynov et al. [26] tried to avoid artifacts for virtual reality applications and they measured the quality of a depth map upsampling using renderings of the resulting 3D surfaces.

In recent years, there have been a lot of remarkable works in single-image super-resolution (SISR) tasks, which have common ground with our depth map reconstruction task. Lim et al. [27] developed a multi-scale deep SR system that can reconstruct HR images of different upscaling factors in a single model. Zhang et al. [28] proposed a residual dense network that uses a residual dense block to extract local features with a contiguous memory mechanism and then learned global hierarchical features by fusing dense local features jointly and adaptively. Zhang et al. [1] proposed the very deep residual channel attention networks formed by residuals in a residual structure and a channel attention mechanism such that channel-wise features are treated differently. Liu et al. [29] proposed a kind of non-local module to capture deep feature correlations between each location and its neighborhood and employed the recurrent neural network structure for deep feature propagation. Qiu et al. [30] proposed an embedded block residual network where different modules restore the information of different frequencies for a texture SR. Hu et al. [31] proposed a channel-wise and spatial feature modulation network where LR features can be transformed to high informative features using feature-modulation memory modules. Jing et al. [32] took the LR image and its downsampled resolution (DR) and upsampled resolution (UR) versions as inputs and learned the internal structure coherence with the pairs of UR-LR and LR-DR to generate a hierarchical dictionary. In addition to SISR, multi-image super-resolution (MISR) has gained attention and there have already been some deep learning methods focusing on it. Haris el at. [33] proposed a recurrent backprojection network (RBPN) that integrates spatial and temporal contexts from continuous video frames using a recurrent encoder–decoder module that fuses multi-frame information with a single-frame SR method for the target frame. Molini et al. [34] proposed a CNN-based technique called DeepSUM to exploit spatial and temporal correlations for the SR of a remote sensing scene from multiple unregistered LR images. DeepSUM has three stages including shared 2D convolutions to extract high-dimensional features from the inputs, a subnetwork proposing registration filters, and 3D convolutions for the slow fusion of the features. DeepSUM++ [35] evolved from DeepSUM and shows that non-local information in a CNN can exploit self-similar patterns to provide the enhanced regularization of SR.

## 3. Proposed Dual-Branch Multi-Scale Residual Network with Channel Interaction

In this study, we supposed that an LR depth map $D_{l}$ is obtained by downsampling its corresponding target HR depth map $D_{h}$ and an HR RGB image $Y_{h}$ of the same scene is available. $Y_{h}$ and $D_{l}$ of the same scene are the inputs of our network, and the goal is to reconstruct and output $D_{h}$ end to end at an upscaling factor s.

**Figure 1:**
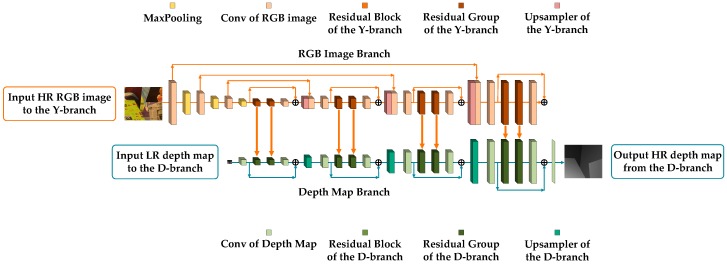
The architecture of our network for 8× upsampling. HR: High-resolution, LR: Low-resolution.

In the following, we take s=8 as an example to show our network structure (see Figure 1).

### 3.1. RGB Image Network Branch

The main role of the RGB image network branch is to provide guidance for the feature map extraction of the deep map network branch. In general, the structure of the Y-branch can be divided into three functional parts. The first part is to downscale the input RGB image by a factor of 2 through a convolution layer and a maxpooling layer for m=log(s) times until the resolution of the feature maps is the same as the input depth map (see Figure 1). Since the sample network has an upscaling factor of 8, such a downsampling operation is executed three times in total. The feature maps obtained in the first part can be expressed as follows:

(1) $$F_{DW\left(1\right)}^{Y}=W_{DW\left(1\right)}^{Y}*Y_{h}+b_{DW\left(1\right)}^{Y}$$

(2) $$F_{DW\left(i\right)}^{Y}=W_{DW\left(i\right)}^{Y}*F_{DW\left(i-1\right)}^{Y}+b_{DW\left(i\right)}^{Y}$$

(3) $$F_{DW\left(2i^{\prime }\right)}^{Y}=\mathrm{MaxPool}\left(F_{DW\left(2i^{\prime }-1\right)}^{Y}\right)$$

where i = {3, 5, …, 2m − 1}, $i^{\prime }$ = {1, 2, …, m}. The operator ∗ represents convolution and $W_{DW}^{Y}$ is a kernel of size 3×3 and $b_{DW}^{Y}$ is a bias vector. The superscript Y means that features or blobs belong to the Y-branch and subscript DW stands for the whole downscaling part.

**Figure 2:**
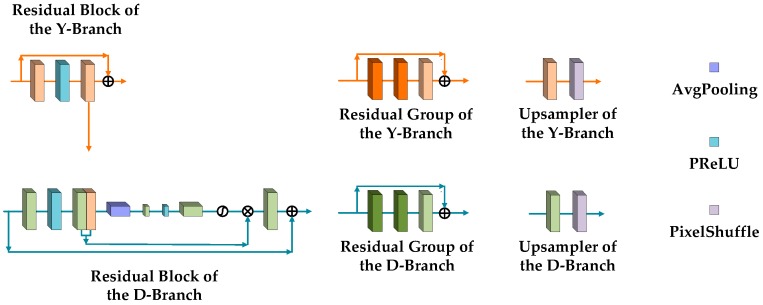
The structure of residual block, residual group and upsampler.

The second part is the parallel network structure matching with the D-branch, which includes a nested structure of residual blocks, groups, and levels. As the most basic constituent unit in the network structure, the residual block of the Y-branch matches with the residual block at the same location in the D-branch. Despite this one-to-one relationship, the residual block for feature extraction in the Y-branch consists of two convolution layers and one PReLU (Parametric Rectified Linear Unit) layer, which is simpler relative to that in the D-branch. After the second convolution operation in the block, the generated feature maps are input into the matched residual block in the D-branch and concatenate feature maps of the depth map guided from the RGB image. In addition, the input feature maps of each residual block are added to the feature maps obtained after feature extraction, which is called a short-skip connection inside the block. Based on the residual block, a residual group is composed of several connected residual blocks and one convolution layer. Similar to a short-skip connection, a long-skip connection is implemented by adding the input and output of each residual group. In the same way, several residual groups and one convolution layer are connected to constitute a residual level and a long-skip connection is also realized in each level using the same addition of input and output. Figure 2 shows the structure of a residual block and a residual group in the Y-branch. The feature maps generated by each residual level l can be expressed as follows:

(4) $$F_{DF\left(1\right)}^{Y}=H_{DF\left(1\right)}^{Y}\left(F_{DW\left(2m\right)}^{Y}\right)$$

(5) $$F_{DF\left(l\right)}^{Y}=H_{DF\left(1\right)}^{Y}\left(F_{UP\left(l-1\right)}^{Y}\right)$$

where l = {2, 3, …, m + 1}. $H_{DF}^{Y}\left(\cdot \right)$ donates the deep feature extraction and $F_{UP}^{Y}$ represents the feature maps from the third part of the Y-branch. In each residual level l, the feature maps generated by each group g can be expressed as follows:

(6) $$F_{l,1}^{Y}=H_{l,1}^{Y}\left(F_{l,0}^{Y}\right)$$

(7) $$F_{l,g}^{Y}=H_{l,g}^{Y}\left(F_{l,g-1}^{Y}\right)$$

(8) $$F_{DF\left(l\right)}^{Y}=F_{l,0}^{Y}+W_{l}^{Y}F_{l,G}^{Y}$$

where g = {2, 3, …, G}, and G is the number of residual groups in a level. $F_{l,0}^{Y}$ is the input of the residual level. $H_{l,g}^{Y}\left(\cdot \right)$ donates the function of the gth residual group. $F_{l,g-1}^{Y}$ and $F_{l,g}^{Y}$ are the input and output of gth residual group, respectively. $W_{l}^{Y}$ is the weight set of the tail convolution layer. In each residual group g, the feature maps generated by each residual block b can be expressed as follows:

(9) $$F_{g,1}^{Y}=H_{g,1}^{Y}\left(F_{g-1}^{Y}\right)$$

(10) $$F_{g,b}^{Y}=H_{g,b}^{Y}\left(F_{g,b-1}^{Y}\right)$$

(11) $$F_{g}^{Y}=F_{g-1}^{Y}+W_{g}^{Y}F_{g,B}^{Y}$$

where b = {2, 3, …, B}, and B is the number of residual blocks in a group. $F_{g-1}^{Y}$ and $F_{g}^{Y}$ are the input and output of gth group, respectively. $H_{g,b}^{Y}\left(\cdot \right)$ donates the function of the bth residual block. $F_{g,b-1}^{Y}$ and $F_{g,b}^{Y}$ are the input and output of the bth residual block, respectively. $W_{g}^{Y}$ is the weight set of the tail convolution layer. In each residual block b, the basic operations can be expressed as follows:

(12) $$h\left(F_{b}^{Y}\right)=W_{b,2}^{Y}*\left(\sigma \left(W_{b,1}^{Y}*F_{b-1}^{Y}+b_{b,1}^{Y}\right)\right)+b_{b,2}^{Y}$$

(13) $$F_{b}^{Y}=F_{b-1}^{Y}+h\left(F_{b}^{Y}\right)$$

where h(⋅) denotes the high-frequency feature maps of the input. σ(⋅) donates the activation function PReLU. $F_{b-1}^{Y}$ and $F_{b}^{Y}$ are the input and output of the bth residual block, respectively. $W_{b,1}^{Y}$ and $W_{b,2}^{Y}$ are kernels of size 3×3, and $b_{b,1}^{Y}$ and $b_{b,2}^{Y}$ are the bias vectors.

The third part of the Y-branch is the resolution enlarging level. This part consists of an upsampler and a convolution layer, and all these layers are connected after the residual level. The upsampler here is composed of a convolution layer and a pixel-shuffling layer. Corresponding to the initial downscaling steps, feature maps are upscaled by a factor of 2 after each residual level and resolution enlarging level. Furthermore, the feature maps from the first part concatenate the feature maps that have the same resolution after upsampling, and then perform a convolution operation (see Figure 2). This design means the upsampled feature maps become supplemented by feature maps with an original high resolution from the first part such that more structured features at different scales can be retained in the network for the processing that follows, meaning that enough guidance is provided to the D-branch. The feature maps generated by the third part can be expressed as follows:

(14) $$F_{UP\left(l^{\prime }\right)}^{Y}=W_{l^{\prime },2}^{Y}*\left(\mathrm{PixelShuffle}\left(W_{l^{\prime },1}^{Y}*F_{DF\left(l^{\prime }\right)}^{Y}+b_{l^{\prime },1}^{Y}\right),F_{DW\left(2m-2l^{\prime }+1\right)}^{Y}\right)+b_{l^{\prime },2}^{Y}$$

where $l^{\prime }$ = {1, 2, …, m}. $W_{l^{\prime },1}^{Y}$ and $W_{l^{\prime },2}^{Y}$ are kernels of size 3×3, and $b_{l^{\prime },1}^{Y}$ and $b_{l^{\prime },2}^{Y}$ are the bias vectors.

Referring to Shi et al. [36], the pixel-shuffling layer rearranges the elements of a $H\times W\times C\cdot r^{2}$ blob B to a blob of shape rH×rW×C, where r is the upscaling factor and H×W is the size of C feature maps. Mathematically, the pixel-shuffling operation can be described as follows:

(15) $$\mathrm{PixelShuffle}{\left(B\right)}_{x,y,c}=B_{⎣x/r⎦,⎣y/r⎦,C\cdot r\cdot \mathrm{mod}\left(y,r\right)+C\cdot \mathrm{mod}\left(x,r\right)+c}$$

where *x* and *y* are the output pixel coordinates of the cth feature map in HR space. The feature maps from the LR space are built into HR feature maps through the pixel-shuffling layer.

### 3.2. Depth Map Network Branch

The task of the depth map network branch is to complete the super-resolution of an LR depth map under guidance from the parallel Y-branch. Compared to the Y-branch, due to the low resolution of the input depth map, the D-branch is mainly composed of two parts, the residual levels and the resolution enlarging levels, without the downscaling part. Except for this difference in architecture, the nested structure of the residual blocks, groups, and the short- or long-skip connections in the D-branch still exist as in the Y-branch. However, the composition of the residual block that contains convolution layers, PReLU layers, and average-pooling layer in the D-branch is more complicated than that in the Y-branch. The whole feature extraction procedure of this kind of residual block is explained as follows. The input feature maps are processed using convolution, PReLU, and convolution first, and then the feature maps from the Y-branch are concatenated. After the subsequent average pooling, convolution, PReLU, convolution again, and applying the sigmoid function, the weights are generated and multiplied by the previous concatenated feature maps to generate new feature maps that not only integrate the structure information coming from the RGB image, but also prevent unreasonable textures from appearing. In addition to these internal structures, the short-skip connection still exists and adds the input and the output of each residual block. Figure 2 shows the structure of the residual block and residual group in the D-branch. The feature maps generated by each residual level l can be expressed as follows:

(16) $$F_{DF\left(1\right)}^{D}=H_{DF\left(1\right)}^{D}\left(W_{0}^{D}*D_{l}+b_{0}^{D}\right)$$

(17) $$F_{DF\left(l\right)}^{D}=H_{DF\left(l\right)}^{D}\left(F_{UP\left(l-1\right)}^{D}\right)$$

where l = {2, 3, …, m + 1}. The superscript D means that features or blobs belong to the D-branch. $W_{0}^{D}$ and $b_{0}^{D}$ are a kernel of 3×3 and a bias vector to the head convolution layer for initial feature extraction, respectively. $H_{DF}^{D}\left(\cdot \right)$ denotes the deep feature extraction and $F_{UP}^{D}$ represents the feature maps from the second part of the D-branch. In each residual level l, the feature maps generated by each group g can be expressed as follows:

(18) $$F_{l,1}^{D}=H_{l,1}^{D}\left(F_{l,0}^{D}\right)$$

(19) $$F_{l,g}^{D}=H_{l,g}^{D}\left(F_{l,g-1}^{D}\right)$$

(20) $$F_{DF\left(l\right)}^{D}=F_{l,0}^{D}+W_{l}^{D}F_{l,G}^{D}$$

where g = {2, 3, …, G}, and G is the number of residual groups in a level. $F_{l,0}^{D}$ is the input of the residual level. $H_{l,g}^{D}\left(\cdot \right)$ denotes the function of the gth residual group. $F_{l,g-1}^{D}$ and $F_{l,g}^{D}$ are the input and output of the gth residual group, respectively. $W_{l}^{D}$ is the weight set of the tail convolution layer. In each residual group g, the feature maps generated by each residual block b can be expressed as follows:

(21) $$F_{g,1}^{D}=H_{g,1}^{D}\left(F_{g-1}^{D}\right)$$

(22) $$F_{g,b}^{D}=H_{g,b}^{D}\left(F_{g,b-1}^{D}\right)$$

(23) $$F_{g}^{D}=F_{g-1}^{D}+W_{g}^{D}F_{g,B}^{D}$$

where b = {2, 3, …, B}, and B is the number of residual blocks in a group. $F_{g-1}^{D}$ and $F_{g}^{D}$ are the input and output of the gth group, respectively. $H_{g,b}^{D}\left(\cdot \right)$ denotes the function of the bth residual block. $F_{g,b-1}^{D}$ and $F_{g,b}^{D}$ are the input and output of the bth residual block, respectively. $W_{g}^{D}$ is the weight set of the tail convolution layer. In each residual block b, the basic operations can be expressed as follows:

(24) $$h\left(F_{b}^{D}\right)=W_{b,2}^{D}*\left(\sigma \left(W_{b,1}^{D}*F_{b-1}^{D}+b_{b,1}^{D}\right)\right)+b_{b,2}^{D}$$

(25) $$F_{b}^{D}=F_{b-1}^{D}+R_{b}^{D}\left(h\left(F_{b}^{D}\right),h\left(F_{b}^{Y}\right)\right)\cdot \left(h\left(F_{b}^{D}\right),h\left(F_{b}^{Y}\right)\right)$$

where h(⋅) denotes the high-frequency feature maps of the input. σ(⋅) denotes the activation function PReLU. $F_{b-1}^{D}$ and $F_{b}^{D}$ are the input and output of the bth residual block, respectively. $W_{b,1}^{D}$ and $W_{b,2}^{D}$ are kernels of size 3×3, and $b_{b,1}^{D}$ and $b_{b,2}^{D}$ are the bias vectors. $R_{b}^{D}\left(\cdot \right)$ denotes the function of the channel interaction.

Except for the difference in the residual block, the D-branch directly employs the upsampler and the convolution layer as a resolution enlarging level to upscale the feature maps without concatenating feature maps from the branch itself due to the lack of a downscaling part. The resolution enlarging level is arranged to be connected after the residual level, which is one of the steps used to gradually achieve super-resolution. Finally, a convolution layer is connected after the last residual layer to convert the feature maps into a depth map to generate a target HR depth map as the whole dual-branch network’s output (see Figure 2). The feature maps generated by the second part can be expressed as follows:

(26) $$F_{UP\left(l^{\prime }\right)}^{D}=W_{l^{\prime },2}^{D}*\mathrm{PixelShuffle}\left(W_{l^{\prime },1}^{D}*F_{DF\left(l^{\prime }\right)}^{D}+b_{l^{\prime },1}^{D}\right)+b_{l^{\prime },2}^{D}$$

where $l^{\prime }$ = {1, 2, …, m}. $W_{l^{\prime },1}^{D}$ and $W_{l^{\prime },2}^{D}$ are kernels of size 3×3, and $b_{l^{\prime },1}^{D}$ and $b_{l^{\prime },2}^{D}$ are the bias vectors.

At the end of our network is a convolution layer that reconstructs feature maps into an output HR depth map ${\tilde{D}}_{h}$ as follows:

(27) $${\tilde{D}}_{h}=W_{REC}^{D}*F_{DF\left(m+1\right)}^{D}+b_{REC}^{D}$$

where $W_{REC}^{D}$ is a kernel of size 3×3, and $b_{REC}^{D}$ is the bias vector.

Our network is optimized with a loss function *L*_1_. Given a training set ${\left\{Y_{h}^{i},D_{l}^{i},D_{h}^{i}\right\}}_{i=1}^{N}$, which contains N HR RGB images and LR depth maps as inputs, along with their HR depth map counterparts, our network is trained by minimizing the *L*_1_ loss function

(28) $$L\left(\Theta \right)=\frac{1}{N}\overset{N}{\underset{i=1}{\sum }}\|{\tilde{D}}_{h}^{i}-D_{h}^{i}{\|}_{1}$$

where Θ denotes the parameter set of our network. This *L*_1_ loss function is optimized using a stochastic gradient descent.

### 3.3. Channel Interaction

Channel attention is a channel-wise feature interaction and change mechanism proposed by Zhang et al. [1], whose goal is to allow the network to pay more attention to features that contain more information. This mechanism originates from two points. One is that there are abundant low-frequency and valuable high-frequency components in LR space. The low-frequency components are mostly flat, and the high-frequency components are mostly regions full of details, such as edges and textures. Another is that each filter of the convolution layer has a local receptive field such that convolution fails to use contextual information outside the local region. In response to these two points, the channel attention mechanism uses global average pooling to obtain channel-wise global spatial information and employs a gating mechanism to capture the dependencies between channels. This gating mechanism can not only learn nonlinear interactions, but also avoids mutual exclusion between channel-wise features. The coefficient factors learned by the gating mechanism are the weights for rescaling the channels. The channel attention mechanism operates between the channel-wise features learned from the input image. We further extended this mechanism to the guidance from the RGB image to the depth map, which makes the features learned by dual-network branches interact with each other.

There are two types of channel interactions in our network. The first one is the concatenation of the feature maps before downscaling and after upsampling in the Y-branch, and then executing the convolution operation for new channel-wise feature maps. This is a relatively common channel-wise interaction procedure, which guarantees that the feature maps of all the channels affect each other equally. The reason for adopting this kind of equal channel interaction is that due to the beginning downscaling part, the loss of details in the previous residual level needs to be supplemented for feature extraction and network learning of the next residual level at a larger scale. Furthermore, the supplemented feature maps also help the guidance provided for the D-branch. The second way channel interaction occurs is through the weight of each channel, which is calculated through a series of functions and decides the influence of its channel in the process of generating new feature maps after the feature maps of each residual block in the D-branch concatenates the feature maps from the Y-branch. The guidance from the Y-branch to the D-branch is realized in this way for the channels from the Y-branch, which can affect all the channels in the residual block. However, each channel from the Y-branch has an unequal influence and interacts with each other according to different weights such that the structured features that have a corresponding relationship between the RGB image and depth map are emphasized and the inconsistent features without such a relationship suppressed. Small weights limit the appearance of artifacts introduced by the feature maps from the Y-branch.

As $R_{b}^{D}\left(\cdot \right)$ denotes the entire operation of channel interaction, we suppose that $X=\left[x_{1}^{Y},\ldots ,x_{c}^{Y},\ldots ,x_{C}^{Y},x_{1}^{D},\;\ldots ,x_{c}^{D},\ldots ,x_{C}^{D}\right]$ is an input, which has C feature maps with a size of H×W from the Yth and Dth branches separately. The channel-wise statistic $z\in {ℜ}^{2C}$ can be obtained by shrinking X, and the cth element of z is:

(29) $$z_{c}=\mathrm{AveragePool}\left(x_{c}\right)=\frac{1}{H\times W}\overset{H}{\underset{h=1}{\sum }}\overset{W}{\underset{w=1}{\sum }}x_{c}\left(h,w\right)$$

where $x_{c}\left(h,w\right)$ is the value at position (h,w) of the cth feature $x_{c}$ from either the Yth or Dth branch. Therefore, we obtain the weight coefficient using the function:

(30) $$s=f\left(W_{U}^{D}\sigma \left(W_{D}^{D}z\right)\right)$$

where f(·) and σ(·) denote the sigmoid and PReLU functions, respectively. $W_{D}^{D}$ is the weight set of a convolution layer that downscales channels with a reduction ratio r. In our experiments, r was set to 16. $W_{U}^{D}$ is also a weight set of a convolution layer that upscales channels with the same ratio r. Then, we can rescale $x_{c}$ by:

(31) $${\hat{x}}_{c}=s_{c}\cdot x_{c}$$

## 4. Evaluation

### 4.1. Network Training

The data set for experiments in this paper was the same as in Hui et al. [23], which consisted of 58 RGBD images from the MPI (Max-Planck Institute) Sintel depth dataset and 34 RGBD images from the Middlebury dataset. Among them, a total of 82 RGBD images made up the training set for our network training, and the other 10 images composed the test set for validation. Our experiments included SR reconstruction of an LR depth map with upscaling factors of 2, 3, 4, 8, and 16 separately. Considering that a factor of 2 was the initial base, we first trained a network with an upscaling factor of 2 whose Y-branch was pre-trained using 1000 images from the NYUv2 (New York University Version 2) dataset [37]; then, the entire network was trained using these 1000 RGB images and depth maps, and finally, the aforementioned training dataset containing 82 RGBD images were used for network fine-tuning. Based on the trained network with an upscaling factor of 2, other networks with upscaling factors of 3, 4, 8, and 16 were further fine-tuned using the same 82 RGBD images.

In terms of the details of training, we gathered LR depth maps to form the training dataset at different upscaling factors by downscaling the corresponding HR depth maps through bicubic interpolation. In the process of training, we did not input large-size images or depth maps into our network directly, but split each one into small overlapping patches and did some common data enhancement before a patch entered the network. The size of these patches was set according to the upscaling factor. The upscaling factors were {2,3,4,8,16}, the corresponding size of the input depth map’s patch were $\left\{48^{2},48^{2},48^{2},24^{2},12^{2}\right\}$, and the sizes of the input RGB image’s patch were $\left\{96^{2},144^{2},192^{2},192^{2},192^{2}\right\}$. Furthermore, the other settings of the network training included the choice of the loss function, optimizer, learning rate, etc. We chose *L*_1_ as the loss function, used the ADAM optimizer where $P_{1}=0.8$, $P_{2}=0.999$, $\varepsilon =10^{-8}$ and the initial learning rate was set to $10^{-4}$. The learning rate was halved after every 200 epochs. We trained all these network models using PyTorch on a GTX 1080 GPU.

### 4.2. Evaluation on the Middlebury Dataset

In order to compare our method with the experimental results of other studies, we used the root mean squared error (RMSE) as an evaluation criterion. Referring to Hui et al. [23], we evaluated our algorithm using Middlebury RGBD datasets whose holes were filled. The dataset was divided into three sets, namely *A*, *B*, and *C*. Data in the table came from References [2,3,6,10,12,13,14,16,17,18,23,24,25]. At each upscaling factor, the best RMSE result of all the algorithms listed in the table is in bold and the sub-optimal result is underlined. For dataset *C*, the comparison was only performed until the upscaling factor increased to 8 because the resolution of the input depth map was too low to reconstruct the HR depth map when the upscaling factor was 16. In addition, the experimental results at the upscaling factor of 3 were not put into the three tables because the other algorithms cannot reconstruct depth maps at a factor that is not a power of 2.

Table 1, Table 2 and Table 3 are records of the evaluation on sets *A*, *B*, and *C* separately, and our algorithm showed an excellent performance compared with the others. When the upscaling factor was small, the gap between the algorithms was not huge, but the advantage of our method was obvious with after increasing the upscaling factor. This phenomenon shows that it is feasible to use an HR RGB image to guide an LR depth map super-resolution in a multi-scaled way if the LR depth map has poor quality and lacks high-frequency information. This condition is a challenge to all the image SR methods. Since References [23,24] adopt a multi-scale mechanism and References [24,25] are built on a residual structure, we focused on the comparison of the experiment results between theirs and ours. According to Table 1, the average RMSE of our network on dataset *A* at the upscaling factors of {2, 4, 8, 16} were {0.37, 0.78, 1.27, 1.89}, which outperformed Hui et al. [23] with gains of {0.09 (+19.6%), 0.15 (+16.1%), 0.23 (+15.3%), 0.71 (+27.3%)}, outperformed Zuo et al. [24] with gains of {0.15 (+28.8%), 0.22 (+22.0%), 0.35 (+21.6%), 0.73 (+27.9%)} and outperformed Zuo et al. [25] with gains of {0.06 (+14.0%), 0.15 (+16.1%), 0.28 (+18.1%), 0.61 (+24.4%)}. On dataset *B*, our network outperformed Hui et al. [23] with gains of {0.07 (18.4%), 0.13 (+15.9%), 0.32 (+22.2%), 0.75 (+31.5%)}, outperformed Zuo et al. [24] with gains of {0.31 (+50%), 0.39 (+36.1%), 0.56 (+33.3%), 1.2 (+42.4%)}, and outperformed Zuo et al. [25] with gains of {0.21 (+40.4%), 0.31 (+31%), 0.51 (+31.3%), 1.09 (+40.1%)}. On dataset *C*, our network outperformed Hui et al. [23] with gains of {0.35 (+38.9%), 0.53 (+24.3%), 0.96 (23.3%)} at the upscaling factors of {2, 4, 8}. Overall, our network substantially reduced the RMSE using these three datasets in the mean sense compared with other methods. Although our network only had sub-optimal results in several cases, such as for Venus in dataset *C*, it is still reasonable to infer that special optimization may be required for some isolated samples.

**Figure 3:**
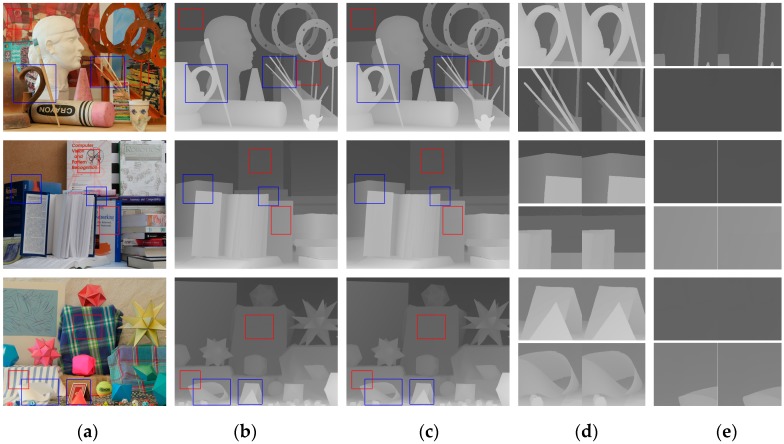
Upsampled depth maps for dataset *A* with an upscaling factor of 8. (**a**) HR RGB images for input, (**b**) ground-truth HR depth maps, (**c**) upsampled results from our network, (**d**) regions inside blue boxes from (**b**) (left) and (**c**), and (**e**) regions inside red boxes from (**b**) (left) and (**c**).

Figure 3 shows the results of our network on dataset *A* with an upscaling factor of 8. To further verify the effectiveness of the network structure we designed, we selected several regions full of details in each HR depth map to observe the differences between our SR results and the ground truths. We examined the effect of our network in terms of two aspects. One aspect was concerned with whether the regions containing edges were blurred after super-resolution. In Figure 3, we marked these regions with blue boxes in (a–c), and give the contrast between the ground truths and our SR results in (d). It is obvious that edges in our SR results were as sharp as those in the ground truths. Generally, deeper networks like ours can learn more complex and finer features, including edges. On the other hand, we examined whether the artifacts existed in the reconstructed HR depth maps. We marked the regions containing textures in the HR RGB image but were complanated in the corresponding HR depth map with red boxes. The contrasts between the reconstructed results and ground truths given in (e) demonstrate that artifacts disappeared after super-resolution. From these results, we can conclude that our proposed method can perform finer depth map SR reconstruction while suppressing the introduction of artifacts.

### 4.3. Evaluation of Generalization

**Figure 4:**
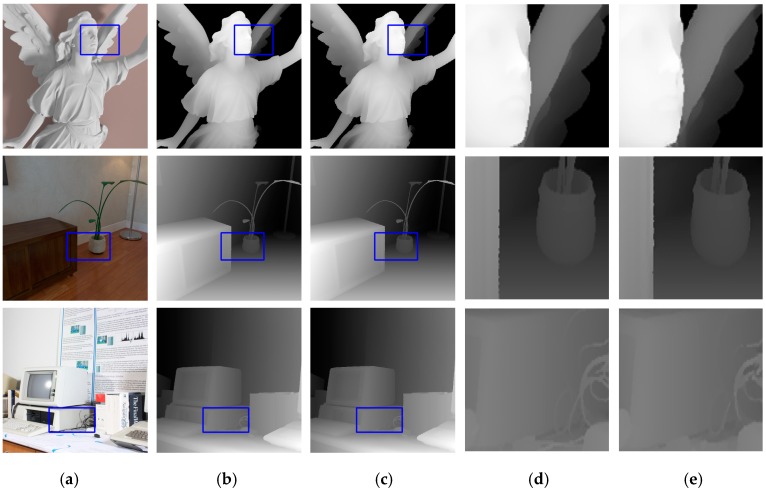
Upsampled depth maps for dataset *Mixture* with an upscaling factor of 4. (**a**) HR RGB images for input, (**b**) ground-truth HR depth maps, (**c**) upsampled results from our network, (**d**) regions inside blue boxes from (**b**), and (**e**) regions inside blue boxes from (**c**).

To test the generalization of our proposed network, we selected three RGBD images from different databases to form a new dataset *Mixture* in which image Lucy from the SimGeo dataset [26], image Plant from the ICL-NUIM (Imperial College London- National University of Ireland Maynooth) dataset [38], and image Vintage from Middlebury dataset were considered. The model we used for evaluation was the same as the model tested on datasets *A*, *B*, and *C* without fine-tuning, and the evaluation criterion was still the RMSE. We mainly tested our method at the upscaling factors of 4 and 8, in comparison with methods from References [23,26,39,40,41]. Our method produced the best performance on the image from the Middlebury dataset and performed nearly 20% better than the sub-optimal result (see Table 4). On the ICL-NUIM dataset, our method’s performance was similar to other methods. However, the results on image Lucy indicated that our network was not suitable for this dataset, which means the generalization ability of our network needs to be improved in the future. Figure 4 shows the results of our network on dataset *Mixture* with an upscaling factor of 4. Details in blue boxes were enlarged and shown in columns (d) and (e).

In Table 5, we provide the time taken by our network and other methods [6,7,23] to upscale the depth map from different low resolutions to full resolution. The computation time of Hui et al. [23] was calculated by upsampling image Art using dataset *A*, and we completed the same experiment on a GTX 1080 GPU using Python. Bicubic, SRCNN, and VDSR were written in MATLAB and Guo et al. [42] provides information about the average running time.

## 5. Conclusions

We proposed a dual-branch residual network that realizes LR depth map super-resolution with channel interaction and multi-scale residual levels under the guidance of an HR RGB image. In the design of the network structure, we made the residual levels of the RGB image branch and the depth map branch parallel for not only the corresponding feature extraction process, but also the guidance process from the RGB image branch to the depth map branch. Furthermore, the channel interaction via weights avoided introducing artifacts into the upscaled depth map. Using a multi-scale method for upscaling the LR depth map helped to alleviate the blur of the HR depth map that is caused by upsampling to a high resolution in one step. The experiments showed that our method performed excellently compared with other methods, especially when the upscaling factor was large. In the future, we hope to explore other methods for the channel-wise feature fusion and go further in the residual network design. In addition, the RGB image branch, as an auxiliary role in our network, has more layers than the depth map branch, which gives room for improved performance regarding compressing the layers of the whole network.

## Figures and Tables

**Table 1:** Quantitative comparison (in RMSE) on dataset *A*.

Method Used	Art				Books				Moebius			
	2x	4x	8x	16x	2x	4x	8x	16x	2x	4x	8x	16x
Bilinear	2.83	4.15	6.00	8.93	1.12	1.67	2.39	3.53	1.02	1.50	2.20	3.18
Narayanan [2]	2.76	3.10	3.51	–	1.17	1.24	1.82	–	0.99	1.03	1.76	–
MRFs [12]	3.12	3.79	5.50	8.66	1.21	1.55	2.21	3.40	1.19	1.44	2.05	3.08
Park et al. [13]	2.83	3.50	4.17	6.26	1.09	1.53	1.99	2.76	1.06	1.35	1.80	2.38
Guided [10]	2.93	3.79	4.97	7.88	1.16	1.57	2.10	3.19	1.10	1.43	1.88	2.85
Kiechle et al. [18]	1.25	2.01	3.23	5.77	0.65	0.92	1.27	1.93	0.64	0.89	1.27	2.13
Ferstl et al. [14]	3.03	3.79	4.79	7.10	1.29	1.60	1.99	2.94	1.13	1.46	1.91	2.63
Lu et al. [3]	–	–	5.80	7.65	–	–	2.73	3.55	–	–	2.42	3.12
SRCNN [6]	1.13	2.02	3.83	7.27	0.52	0.94	1.73	3.10	0.54	0.91	1.58	2.69
MSF [16]	3.01	3.70	4.66	6.68	1.25	1.63	2.02	2.84	1.13	1.51	2.06	2.93
Hui et al. [23]	0.66	1.47	2.46	4.57	0.37	0.67	1.03	1.60	0.36	0.66	1.02	1.63
MFR-SR [24]	0.71	1.54	2.71	4.35	0.42	0.63	1.05	1.78	0.42	0.72	1.10	1.73
RDN-GDE [25]	0.56	1.47	2.60	4.16	0.36	0.62	1.00	1.68	0.38	0.69	1.05	1.65
Ours	**0.44**	**1.17**	**1.96**	**3.24**	**0.35**	**0.60**	**0.96**	**1.24**	**0.32**	**0.58**	**0.89**	**1.18**

**Table 2:** Quantitative comparison (in RMSE) on dataset *B*.

Method Used	Dolls				Laundry				Reindeer			
	2x	4x	8x	16x	2x	4x	8x	16x	2x	4x	8x	16x
Bicubic	0.91	1.31	1.86	2.63	1.61	2.41	3.45	5.10	1.94	2.81	3.99	5.82
Narayanan [2]	0.84	1.25	1.69	–	1.34	1.87	2.65	–	1.79	2.02	2.40	–
Park et al. [13]	0.96	1.30	1.75	2.41	1.55	2.13	2.77	4.16	1.83	2.41	2.99	4.29
Ferstl et al. [14]	1.12	1.36	1.86	3.57	1.99	2.51	3.76	6.41	2.41	2.71	3.79	7.27
Kiechle et al. [18]	0.70	0.92	1.26	1.74	0.75	1.21	2.08	3.62	0.92	1.56	2.58	4.64
AP [17]	1.15	1.35	1.65	2.32	1.72	2.26	2.85	4.66	1.80	2.43	2.95	4.09
SRCNN [6]	0.58	0.95	1.52	2.45	0.64	1.18	2.43	4.58	0.77	1.50	2.86	5.25
MSF [16]	1.15	1.43	1.80	2.49	1.93	2.37	3.18	4.58	2.36	2.76	3.53	4.74
Hui et al. [23]	0.35	0.69	1.05	1.60	0.37	0.79	1.51	2.63	0.42	0.98	1.76	2.92
MFR-SR [24]	0.60	0.89	1.22	1.74	0.61	1.11	1.75	3.01	0.65	1.23	2.06	3.74
RDN-GDE [25]	0.56	0.88	1.21	1.71	0.48	0.96	1.63	2.86	0.51	1.17	2.05	3.58
Ours	**0.27**	**0.64**	**0.99**	**1.34**	**0.34**	**0.64**	**1.06**	**1.50**	**0.33**	**0.78**	**1.31**	**2.04**

**Table 3:** Quantitative comparison (in RMSE) on dataset *C*.

Method Used	Tsukuba			Venus			Teddy			Cones		
	2x	4x	8x	2x	4x	8x	2x	4x	8x	2x	4x	8x
Kiechle et al. [18]	3.65	6.21	10.08	0.61	0.82	1.17	1.20	1.82	2.37	1.47	2.97	4.52
Ferstl et al. [14]	5.25	7.35	–	1.11	1.74	–	1.69	2.60	–	2.19	3.50	–
Lu et al. [3]	–	10.29	13.77	–	1.73	2.13	–	2.72	3.47	–	3.99	5.34
SRCNN [6]	3.28	7.94	11.28	0.46	0.79	1.71	1.17	1.99	3.25	1.48	3.59	5.18
Hui et al. [23]	1.85	4.29	8.43	**0.14**	**0.35**	1.04	0.71	1.49	2.76	0.91	2.60	4.23
Ours	**0.91**	**2.75**	**6.18**	0.21	0.42	**0.95**	**0.55**	**1.34**	**2.16**	**0.51**	**2.09**	**3.33**

**Table 4:** Quantitative comparison (in RMSE) on dataset *Mixture*.

Method Used	Lucy	Plant		Vintage	
	4x	4x	8x	4x	8x
Bicubic	0.27	0.25	0.29	0.26	0.30
PDN [39]	0.25	0.27	0.31	0.32	0.35
SRfS [40]	0.37	0.28	0.31	0.35	0.38
DG [41]	0.25	0.27	0.29	0.29	0.30
Hui [23]	0.26	**0.23**	0.29	0.29	0.36
DIP-V [26]	**0.22**	0.26	**0.28**	0.34	0.44
Ours	0.40	0.24	0.31	**0.19**	**0.24**

**Table 5:** Computation time (seconds).

Method Used	2x	3x	4x	8x	16x
Bicubic	0.01	0.01	0.01	0.01	0.01
SRCNN [6]	46.63	46.55	46.87	–	–
VDSR [7]	0.44	0.44	0.45	0.44	0.47
Hui [23]	0.247	–	0.296	0.326	0.368
Ours	4.17	3.99	5.21	6.72	39.89
